# PWS/AS MS-MLPA Confirms Maternal Origin of 15q11.2 Microduplication

**DOI:** 10.1155/2015/474097

**Published:** 2015-05-07

**Authors:** Angelika J. Dawson, Janice Cox, Karine Hovanes, Elizabeth Spriggs

**Affiliations:** ^1^Cytogenetic Laboratory, HSC, Diagnostic Services of Manitoba, 820 Sherbrook Street, Winnipeg, MB, Canada R3A 1K9; ^2^Departments of Biochemistry & Medical Genetics and Pediatrics & Child Health, University of Manitoba, Winnipeg, MB, Canada R3E 0J9; ^3^Genetics & Metabolism Program, WRHA, Winnipeg, MB, Canada R3A 1R9; ^4^Molecular Diagnostic Laboratory, HSC, Diagnostic Services of Manitoba, 820 Sherbrook Street, Winnipeg, MB, Canada R3A 1K9; ^5^CombiMatrix Diagnostics, 310 Goddard, Suite 150, Irvine, CA 92618, USA

## Abstract

The proximal region of the long arm of chromosome 15q11.2-q13 is associated with various neurodevelopmental disorders, including Prader-Willi (PWS) and Angelman (AS) syndromes, autism, and other developmental abnormalities resulting from deletions and duplications. In addition, this region encompasses imprinted genes that cause PWS or AS, depending on the parent-of-origin. This imprinting allows for diagnosis of PWS or AS based on methylation status using methylation sensitive (MS) multiplex ligation dependent probe amplification (MLPA). Maternally derived microduplications at 15q11.2-q13 have been associated with autism and other neuropsychiatric disorders. Multiple methods have been used to determine the parent-of-origin for 15q11.2-q13 microdeletions and microduplications. In the present study, a four-year-old nondysmorphic female patient with developmental delay was found to have a *de novo* ~5 Mb duplication within 15q11.2 by oligonucleotide genomic array. In order to determine the significance of this microduplication to the clinical phenotype, the parent-of-origin needed to be identified. The PWS/AS MS-MLPA assay is generally used to distinguish between deletion and uniparental disomy (UPD) of 15q11.2-q13, resulting in either PWS or AS. However, our study shows that PWS/AS MS-MLPA can also efficiently distinguish the parental origin of duplications of 15q11.2-q13.

## 1. Introduction

**Figure 1:**
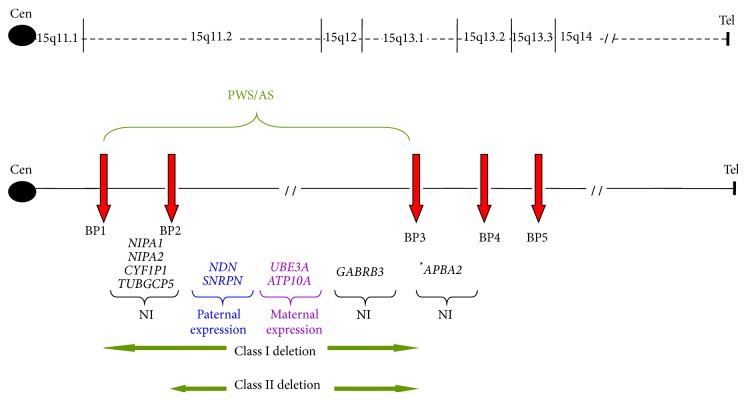
Chromosome 15 ideogram showing breakpoints BP1–BP5 in 15q11.2-q13 and the PWS/AS common deletion regions (green). Genes located in each breakpoint interval are shown below (blue = paternal expression/maternal imprint; purple = maternal expression/paternal imprint; black = biallelic expression/no imprint). ^*^APBA2 is a nonimprinted gene utilized in MS-MLPA (see Figure 2).

Multiple segmental duplications, located at common breakpoints 1–5 (BP1–BP5) in the 15q11.2-q13 region, lead to nonallelic homologous recombination (NAHR), resulting in various deletion and microduplication events [1] (Figure 1). The classic PWS/AS deletions (class I or II) are flanked by either BP1 or BP2 and by the more distal BP3 [1]. The region between BP2 and BP3 contains a cluster of methylated, imprinted genes including* SNRPN* and* UBE3A*, which are either paternally or maternally expressed, respectively, but not both. Deletion or uniparental disomy (UPD) for this region results in either PWS or AS, depending on the parent-of-origin of the abnormal chromosome [2]. The region between BP1 and BP2 contains four evolutionarily conserved, nonimprinted genes (*NIPA1*,* NIPA2*,* CYF1P1*, and* TUGCP5*) and is a susceptibility region associated with neurological dysfunction [1]. Deletions flanked by BP3 and BP4 may contribute to variable abnormal phenotypes including short stature, failure to thrive, microcephaly, and hypotonia [3]. The 15q13.3 microdeletion syndrome occurs between BP4 and BP5 and is associated with a variable phenotype of neurocognitive dysfunction, including intellectual disability, autism, seizures, and psychiatric disorders, with reduced penetrance [4]. Maternal duplications involving 15q11.2-q13 region have been also associated with autism, developmental delay, intellectual handicap, seizures, and hypotonia [5] and are a recognized genetic syndrome [6]. Although the number of cases is small, paternal duplications alone do not appear to be fully penetrant for an autism phenotype [6].

Routine clinical testing for methylated CpG residues includes RFLP using methylation sensitive enzymes followed by Southern blot, methylation specific PCR using bisulfite-treated DNA samples, allele specific PCR followed by gel electrophoresis, methylation sensitive high-resolution melting (MS-HRM) curve analysis [6], and MS-MLPA.

MS-MLPA detects methylation status of CpG residues, as well as gene copy number. The MS-MLPA ME028-B1 PWS/AS probe mixture used in our laboratory was purchased through MRC-Holland and contains five probes for imprinted genes* NDN* (1 probe) and* SNRPN *(4 probes). These probes contain a recognition site for HhaI, a methylation sensitive restriction enzyme used to determine methylation status of the region. The remaining probes are used to detect copy number changes. When a copy number change (CNC) is detected, the methylation status indicates if this CNC is maternal or paternal in origin. If there is no CNC detected, the methylation status differentiates between normal biparental inheritance and UPD. For each patient, genotypes are assigned based on their copy number from MLPA analysis (normalized fluorescence peak ratios between individual probes of a patient sample as compared to a control DNA sample) and their corresponding methylation ratio as determined by MS-MLPA (normalized fluorescence peak ratio between HhaI digested and undigested probes from an imprinted region of a patient sample). A peak ratio (copy number) of 1.0 (two copies) with a methylation ratio of 0.5 (both maternal and paternal alleles) represents a wild type sequence; a peak ratio of 1.0 (two copies) with a methylation ratio of 1 (maternal alleles only) or 0 (paternal alleles only) represents UPD for either PWS or AS, respectively; a peak ratio of 0.5 (one copy) with a methylation ratio of 1 (maternal alleles only) or 0 (paternal alleles only) represents either a PWS or AS deletion, respectively; and a peak ratio of 1.5 (three copies) with a methylation ratio of ~0.7 (2 of 3 maternal alleles) or ~0.3 (2 of 3 paternal alleles) represents either a maternal or paternal duplication, respectively. Although the PWS/AS MS-MLPA kit is used extensively in molecular diagnostic laboratories for PWS/AS analysis, there have been few reports of using this kit for the identification of parent-of-origin for duplications of 15q11.2-q13. Parent-of-origin of proximal 15q duplications using MS-MLPA was determined initially in a cell line from the Coriell Repository (GM12135) [7] and subsequently in patients [8].

## 2. Case Presentation and Discussion

**Figure 2:**
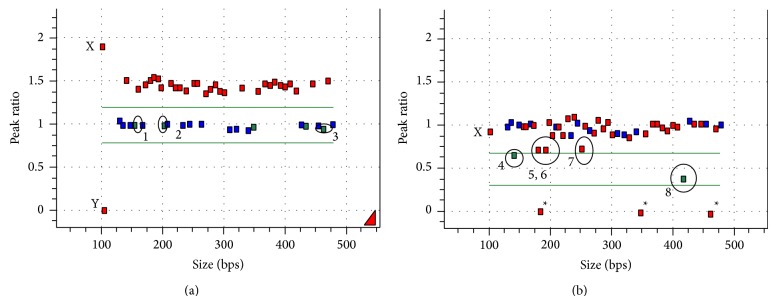
MS-MLPA analysis for determination of 15q11.2 duplication. *x*-axes represent fragment size in bps and *y*-axes represent probe peak ratios. (a) MLPA copy number peak ratio: red = PWS/AS region probes; green = non-PWS/AS region probes. 1 = TUBGCP5; 2 = APBA2; 3 = NIPA1; X (2 copies) and Y (0 copies) chromosome in female patient. (b) MS-MLPA digestion of five HhaI methylation sensitive probes: 4 = SNRPN intron 1; 5 = SNRPN exon 1; 6 = SNRPN intron 1; 7 = SNRPN promoter; 8 = NDN; ∗ = HhaI digestion controls.

In the present study, a four-year-old nondysmorphic female patient with developmental delay was found to have a class II* de novo* ~5 Mb duplication within 15q11.2, between BP2 and BP3, by oligonucleotide 180 K microarray, which was confirmed by FISH: nuc ish 15q11.2(RP11-1071C22x3)dn.arr [hg19] 15q11.2(23,664,484-28,602,810)x3 (CombiMatrix Diagnostics, Irvine, CA). The MLPA analysis showed that all chromosome 15 probes within the PWS/AS region had a peak ratio (copy number) of ~1.5 after ligation and PCR amplification (Figure 2(a)). Probes for the* NIPA1* and* TUBGCP5* genes (circled), located between BP1 and BP2, proximal to the PWS/AS region, and the* APBA2* gene (circled), located between BP3 and BP4 and distal to the PWS/AS region, showed normal copy number of ~1.0. The ligation, digestion, and PCR amplification for four of the five methylation sensitive probes (circled) resulted in a methylation ratio of ~0.7 (Figure 2(b)). The fifth methylation sensitive probe,* NDN*, with a known tendency to overdigest (MRC-Holland), resulted in an apparent decrease of methylation ratio of 0.41. The remaining nonmethylation sensitive probes have a peak ratio of ~1.0. This MS-MLPA pattern suggests that the 15q11.2 duplication in this patient is of maternal origin and the MLPA results demonstrate that this duplication likely represents the reciprocal product leading to the common PWS/AS deletion.

In order to verify the MS-MLPA results, eight unlinked microsatellite markers located on chromosome 15 were analyzed for this proband and her parents. One marker,* GABRB3* (paternal = 193/201; maternal =** 185/197**), confirmed a maternal duplication with one paternal and two maternal alleles (**185/197/**201). The markers distal to* GABRB3* are consistent with normal biparental inheritance and the proximal markers were noninformative. This suggests that the duplication occurred as a result of unequal maternal meiotic exchange and recombination in 15q11.2-q13.

In conclusion, the MS-MLPA assay can not only distinguish between deletion and UPD resulting in either PWS or AS, but can also distinguish the parental origin of duplications of 15q11.2-q13. MS-MLPA can therefore be used as an adjunct technology to elucidate the etiologic mechanism of 15q11.2-q13 rearrangements detected by microarray or karyotype.
